# The Four FAD-Dependent Histone Demethylases of Arabidopsis Are Differently Involved in the Control of Flowering Time

**DOI:** 10.3389/fpls.2019.00669

**Published:** 2019-06-04

**Authors:** Damiano Martignago, Benedetta Bernardini, Fabio Polticelli, Daniele Salvi, Alessandra Cona, Riccardo Angelini, Paraskevi Tavladoraki

**Affiliations:** ^1^Department of Science, Roma Tre University, Rome, Italy; ^2^Centre for Research in Agricultural Genomics, Spanish National Research Council–Institute for Food and Agricultural Research and Technology–Autonomous University of Barcelona–University of Barcelona, Barcelona, Spain; ^3^‘Roma Tre’ Section, National Institute of Nuclear Physics, Rome, Italy; ^4^Department of Life, Health and Environmental Sciences, University of L’Aquila, L’Aquila, Italy

**Keywords:** flowering time, histone demethylases, FLD, FLC, FWA, LDL, LSD1

## Abstract

In *Arabidopsis thaliana*, four FAD-dependent lysine-specific histone demethylases (LDL1, LDL2, LDL3, and FLD) are present, bearing both a SWIRM and an amine oxidase domain. In this study, a comparative analysis of gene structure, evolutionary relationships, tissue- and organ-specific expression patterns, physiological roles and target genes for the four Arabidopsis *LDL/FLDs* is reported. Phylogenetic analysis evidences a different evolutionary history for the four *LDL/FLDs*, while promoter activity data show that *LDL/FLDs* are strongly expressed during plant development and embryogenesis, with some gene-specific expression patterns. Furthermore, phenotypical analysis of loss-of-function mutants indicates a role of all four Arabidopsis *LDL/FLD* genes in the control of flowering time, though for some of them with opposing effects. This study contributes toward a better understanding of the *LDL/FLD* physiological roles and may provide biotechnological strategies for crop improvement.

## Introduction

Histone methylation is involved in a wide range of biological processes (Pfluger and Wagner, 2007). It decorates both transcriptionally silenced and active chromatin domains, depending on which residues are methylated and the degree of methylation. One of the most relevant and studied histone marks in plants is the methylation of lysine 3 on histone 4 (H3K4me). H3K4 can be mono-, di-, and tri-methylated (respectively me1, me2, me3) by different classes of SET domain-containing methyltransferases and this process is reversed by histone demethylases in a dynamic fashion. Two types of lysine-specific histone demethylases are present in both animals and plants, the Jumonji C (JmjC) domain-containing histone demethylases and the FAD-dependent histone demethylases (JHDMs and LSDs, respectively; Shi et al., 2004; Tsukada et al., 2005; Xu et al., 2015; Gu et al., 2016). In animals, two LSDs are found, LSD1 and LSD2 (Shi et al., 2004; Karytinos et al., 2009), which contain a FAD-dependent amine oxidase (AO; Polticelli et al., 2005) domain and a SWIRM domain (Stavropoulos et al., 2006). LSD1 has also an ∼100 amino acid protruding domain, known as ‘Tower’ domain, which interacts with the corepressor CoREST, among other proteins, and is required for LSD1 catalytic activity on nucleosomes (Shi et al., 2005; Chen et al., 2006; Stavropoulos et al., 2006; Yang et al., 2006; Burg et al., 2015). Unlike LSD1, LSD2 does not have the ‘Tower’ domain and does not interact with CoREST, but possesses both a CW-type zinc finger motif and a C4H2C2-type zinc finger motif joined by a linker domain composed of two α-helices. This suggests that LSD2 may interact with different targets or co-regulatory molecules and may be involved in transcriptional programs distinct from those of LSD1 (Burg et al., 2015).

*Arabidopsis thaliana* has four homologs of the human *LSD1* (*HsLSD1*) gene: *At1g6283*0 (*LSD1-LIKE1*; *LDL1*), *At3g13682* (*LDL2*), *At4g16310* (*LDL3*), and *At3g10390* (*FLD*), all bearing both a flavin AO domain and a SWIRM domain (Shi et al., 2004; Jiang et al., 2007; Spedaletti et al., 2008). Like HsLSD1, Arabidopsis LDL1 is able to specifically demethylate H3K4me2 and H3K4me1 peptides and to discriminate between different epigenetic marks (Forneris et al., 2005; Spedaletti et al., 2008). Furthermore, LDL1 interacts with a SET-domain histone methyltransferase and a histone deubiquitinase to form co-repressor complexes (Krichevsky et al., 2007, 2011). However, plant LDL/FLDs are probably directed to their substrates by mechanisms different from those of their animal counterparts (Sadiq et al., 2016). Indeed, plants do not encode CoREST homologs. In addition, LDL/FLDs do not interact with plant homologs of SFMBT1, which functions as part of the LSD1-based repressor complex and is known to bind different forms of methylated histones (Tang et al., 2013; Zhang J. et al., 2013; Sadiq et al., 2016).

Most of the physiological studies on the Arabidopsis LDL/FLDs focus on their role in the control of flowering time. The developmental transition from the vegetative to the reproductive stage is a critical event in the plant life cycle. In *A. thaliana*, a complex regulatory network controls the timing of floral transition, a key component of which is FLOWERING LOCUS C (FLC), a MADS-box transcriptional regulator that inhibits floral transition largely by reducing the expression of flowering-time integrators, such as *SUPPRESSOR OF OVEREXPRESSION OF CONSTANS 1* (*SOC1)* and *FLOWERING LOCUS T* (*FT*) (He et al., 2003; He, 2009). This regulatory network integrates the endogenous developmental state of the plant (autonomous pathway and gibberellin-dependent pathway) with environmental cues (Amasino and Michaels, 2010). FLD is involved in the autonomous pathway by constitutively repressing *FLC*. Indeed, Arabidopsis *fld* loss-of-function mutants are known to be late-flowering or non-flowering due to increased *FLC* expression levels (Sanda and Amasino, 1996; Chou and Yang, 1998; He et al., 2003; Jiang et al., 2007). FLD is also required in chromatin silencing of *FLC* mediated by the RNA-binding protein FCA (Liu et al., 2007). Furthermore, the physical interaction between FLD and the histone deacetylases HDA5 and HDA6 plays an important role in the control of both H3 acetylation and H3K4 trimethylation at *FLC* and its homologs *MADS AFFECTING FLOWERING 1* (*MAF1*), *MAF4* and *MAF5* (Yu et al., 2011; Luo et al., 2015). Indeed, *fld* mutants display altered H3 and H4 acetylation levels at *FLC* (He et al., 2003; Zhang Y. et al., 2013; Hu et al., 2014). *FLC* is down-regulated also by LDL1 and LDL2, which act in partial redundancy with FLD, the latter playing a more prominent role (Jiang et al., 2007). Consistently, *ldl1ldl2* mutants display increased H3K4me3 levels at *FLC* as compared to wild-type plants, but to a lesser degree than *ldl1fld* mutants. LDL1 and LDL2, but not FLD, are additionally involved in the control of H3K4 methylation state at *FWA*, a homeodomain-containing transcription factor which interferes with floral transition (Jiang et al., 2007). Altogether, these data suggest that the Arabidopsis *LDL*/*FLD* gene family plays a critical role in the histone methylation pattern of flowering genes. A similar function was also suggested for LDL/FLD homologs in other plant species (Hu et al., 2014; Gu et al., 2016; Shibaya et al., 2016).

Recent studies have evidenced the involvement of the *LDL*/*FLD* gene family also in several developmental and stress defense processes (Yu et al., 2016). In fact, LDL1 is involved in root elongation and lateral root initiation (Krichevsky et al., 2009; Singh et al., 2012). In addition, LDL1 and LDL2 repress the expression of seed dormancy-related genes and act redundantly in repressing seed dormancy (Zhao et al., 2015). Furthermore, FLD is required for activation of systemic acquired resistance, through a FLC-independent pathway, and for up-regulation of important modulators of plant immune responses (Singh et al., 2013, 2014; Banday and Nandi, 2018). In wheat, a LDL1-homolog is up-regulated in heat-primed plants suggesting a role of this gene family in the epigenetic mechanisms regulating stress memory (Wang et al., 2016).

The increasing evidence for the involvement of the *LDL*/*FLD* gene family in different physiological processes raises the need for a comparative analysis of this gene family. To this end, in the present study the gene and protein structure, as well as the evolutionary history of all four *LDL*/*FLD*s have been dissected. Furthermore, the tissue- and organ-specific expression patterns of the four *LDL*/*FLDs* were analyzed. Phenotypical analyses of loss-of-function mutants for all four *LDL*/*FLD* genes were also performed, with particular attention to the flowering time, revealing functional differences among them.

## Materials and Methods

### Protein Sequence Homology Search and Retrieval

The amino acid sequence of LSD1-like proteins from various plant and animal organisms were retrieved by sequence similarity searches in BLASTP (Altschul et al., 1997) using the amino acid sequence of HsLSD1 and HsLSD2, as well as of the *A. thaliana* LDL1, LDL2, FLD, and LDL3 as query sequences. The amino acid sequence of additional LSD1-like proteins was retrieved from the National Center for Biotechnology Information (NCBI) database based on sequence annotation. Abbreviations and accession numbers are listed in Supplementary Table 1. To determine SWIRM and AO domains, multiple amino acid sequence alignments were performed using Clustal Omega (Sievers et al., 2011). For genomic exon–intron structure comparisons, manual alignment between genomic and cDNA sequences was performed. Information on intron number was additionally obtained from the NCBI database.

### Molecular Modeling

Molecular models of *A. thaliana* LDL3, and LDL3 homologs from *Physcomitrella patens* (PpLDL3) and *Selaginella moellendorffii* (SmLDL3) have been built using the *ab initio*/threading protocol implemented in the I-TASSER pipeline (Yang et al., 2015). No query/template alignment has been provided in input as I-TASSER uses LOMETS (Local Meta-Threading Server) to thread the query sequence through a representative library of PDB structures and select the folds compatible with the sequence of the query protein (Wu and Zhang, 2007). Best models have been selected on the basis of the I-TASSER quality score (C-score) whose values range from -5 to 2, higher values indicating higher quality models (Yang et al., 2015). C-score values of the selected models for Arabidopsis LDL3, SmLDL3 and PpLDL3 are -0.27, 0.86, and 0.6, respectively.

### Phylogenetic Analyses

Amino acid sequences were aligned with MAFFT v.7 (Katoh and Standley, 2013) using the E-INS-i iterative refinement algorithm. Two alignments were built, one with the entire protein sequence, and another one including only amino acids of the AO domain. For each alignment, the optimal model of protein evolution was selected by ModelTest-NG v0.1.5^1^ under the corrected Akaike Information Criterion. The JTT model (Jones et al., 1992) with gamma distributed rates across site (+G) was selected for both alignments. Phylogenetic analyses were performed with the Maximum Likelihood method using RAXML v.8.2.10 (Stamatakis, 2014) with the PROTGAMMAJTT substitution model. Node support was evaluated with 1,000 rapid bootstrap inferences. The sequence of the polyamine oxidase 1 of *A. thaliana* (AtPAO1; At5g13700; Supplementary Table 1) was used as outgroup. Phylogenetic analyses were computed in the CIPRES Science Gateway V. 3.3^2^ (Miller et al., 2010).

### Plant Material

All experiments were performed with Arabidopsis ecotype Columbia-0 plants grown under long-day (16 h day/8 h night) photoperiod conditions. To determine the flowering time (expressed as the number of rosette leaves at bolting), seeds were sown in a 3:1 soil:perlite mixture and plants were grown to mature stage. For RT-PCR and qRT-PCR analyses, seedlings were grown for 7 days on plates containing half-strength Murashige and Skoog basal medium supplemented with Gamborg’s vitamins and 0.5% (w/v) sucrose (½MS) and solidified with 0.7% agar. Then, seedlings were transferred in 6-well plates containing ½MS liquid medium and were left to grow for 7 more days.

### Characterization of Loss-of-Function *LDL*/*FLD* Mutants

Arabidopsis *ldl1*, *ldl2*, and *fld* loss-of-function mutants were obtained from the SALK collection (SALK_142477.31.30.x, SALK_146346.52.50.x, and SALK_015053.35.80.x, respectively; Alonso et al., 2003), while *ldl3* mutant was obtained from the SAIL library (SAIL_640_B10.v1; Sessions et al., 2002). The presence of T-DNA insertion was confirmed by PCR, and homozygous mutant plants were selected. RT-PCR analysis using primers upstream and downstream from the T-DNA insertion confirmed the absence of correct mRNA for the corresponding genes, whereas qRT-PCR analysis confirmed reduced gene-specific expression levels (Supplementary Figure 2). Primer sequences are listed in Supplementary Table 2.

### Construction and Characterization of Arabidopsis Transgenic Plants

To construct *LDL/FLD::GFP-GUS* transgenic Arabidopsis plants, 2- to 3-kb promoter regions including the 5′UTR were amplified from Arabidopsis *g*enomic DNA by PCR and cloned into the pDONR207 vector (Invitrogen) via Gateway Technology (Invitrogen). Sequences of oligonucleotides used for the amplification of promoter regions are shown in Supplementary Table 2. Following sequencing, promoter regions were inserted into the Gateway binary vector pKGWFS7 vector (Karimi et al., 2002) in-frame with the downstream green fluorescent protein (GFP) and β-glucuronidase (GUS) reporter genes. The resulting constructs were used to transform *A. thaliana* wild-type plants by the *Agrobacterium tumefaciens*-mediated floral dip transformation method (Bent, 2006). Independently transformed plant lines were tested by PCR.

### Histochemical GUS Assay

GUS staining of Arabidopsis *LDL/FLD::GFP-GUS* transgenic plants was performed essentially as previously described (Fincato et al., 2012). Briefly, samples were gently soaked in 90% (v/v) cold acetone for 1 h at -20°C, rinsed with 50 mM sodium phosphate buffer pH 7.0, vacuum infiltrated in staining solution (1 mM 5-bromo-4-chloro-3-indolyl-β-D-glucuronide, 2.5 mM potassium ferrocyanide, 2.5 mM potassium ferricyanide, 0.2% Triton X-100, 10 mM EDTA, 50 mM sodium phosphate buffer, pH 7.0) and incubated at 37°C for 18 h. Chlorophyll was extracted with ethanol:acetic acid (3:1). Samples were kept in 70% ethanol. To improve destaining, reproductive organs were washed with Hoyer’s light medium (Stangeland and Salehian, 2002).

### Quantitative RT-PCR Analysis

Total RNA was isolated from whole Arabidopsis seedlings using the RNeasy Plant Mini kit (QIAGEN) and treated with RNase-free DNase during RNA purification (RNase-Free DNase Set; QIAGEN) according to the manufacturers’ protocol. RNA concentration was measured with a NanoDrop ND-1000 UV-Vis spectrophotometer (NanoDrop Technologies). Synthesis of cDNA and PCR amplification were carried out using GoTaq^®^ 2-Step RT-qPCR System (Promega). The qPCR reactions were performed in a Corbett RG6000 (Corbett Life Science, QIAGEN) following the program: 95°C for 2 min then 40 cycles of 95°C for 3 s and 60°C for 30 s. Primers were designed using Primer3 software (Untergasser et al., 2007) and tested for specificity using Primer-BLAST. *UBIQUITIN-CONJUGATING ENZYME 21* (*UBC21*, *At5g25760*) was chosen as a reference gene (Czechowski et al., 2005). Primer sequences are listed in Supplementary Table 2. Relative expression levels are expressed as fold-changes (2^-ΔΔCt^). Reactions were performed in triplicate and mean values ± SE were calculated. At least three independent biological replicates were performed for each experiment, and mean values of relative expression levels from the different biological replicates are shown.

## Results

### The Arabidopsis *LDL*/*FLD* Gene Family

**FIGURE 1:**
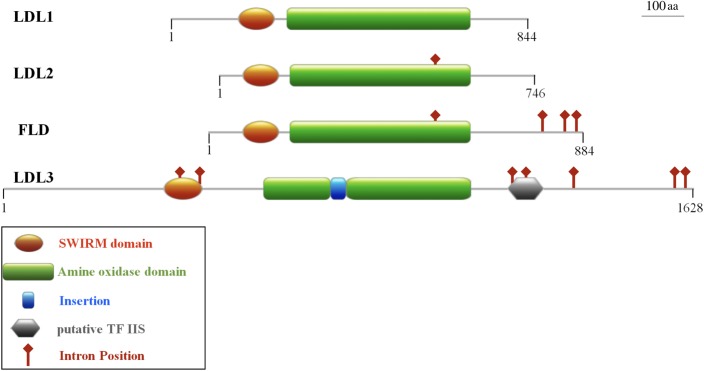
Domain organization of the four Arabidopsis LDL/FLD proteins. All the four proteins contain a SWIRM domain and an amine oxidase domain. LDL3 also presents an insertion in the amine oxidase domain and a putative structured domain with similarity to the transcription factor IIS. Intron positions are shown. Numbers indicate the length of the amino acid primary sequence. The schematic representations are in scale.

The Arabidopsis LDL1, LDL2, and FLD display a high amino acid sequence identity with each other (48–52%; Spedaletti et al., 2008) and a shared gene structure, although with a different number of introns (Figure 1). In particular, *LDL1* gene has no intron, *LDL2* has one and *FLD* four, one of the *FLD* introns at the same position as the single intron in *LDL2* (Figure 1, red diamonds). These similarities suggest that *LDL1*, *LDL2*, and *FLD* are recent derivatives of a common ancestor. In contrast, *LDL3* gene structure is different from that of the other three *LDL/FLD* genes displaying seven introns, all of them at different position with respect to the *FLD* introns (Figure 1). Furthermore, the amino acid sequence identity of LDL3 with the other three LDL/FLDs is low (25–30%). LDL3 amino acid sequence (1,628 amino acids) is also significantly longer than that of the other LDL/FLDs (746–884 amino acids). In particular, LDL3 displays longer *N*-terminal and *C*-terminal extensions, as well as a larger region linking SWIRM and AO domains (SWIRM/AO distance), in respect to those of LDL1, LDL2, and FLD. Interestingly, at the *C*-terminal extension of LDL3, a putative structured domain with some similarity to transcription factor IIS was identified, which may have a regulatory role (Figure 1). DNA-binding domains are also present in the fungal (SWIRM1 and SWIRM2) and HsLSD2 homologs, which display a HMG box and a zinc finger domain, respectively (Nicolas et al., 2006; Zhang Q. et al., 2013).

**FIGURE 2:**
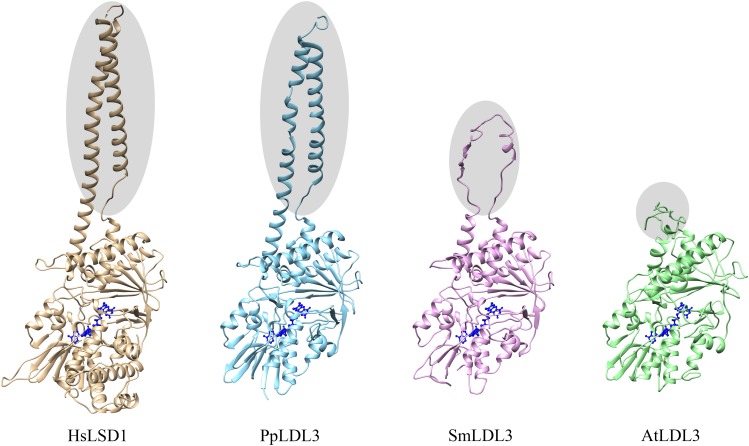
Schematic representation of the three-dimensional structure of human LSD1, Arabidopsis LDL3, as well as *Physcomitrella patens* and *Selaginella moellendorffii* LDL3 homologs. The three-dimensional structure of human LSD1 (HsLSD1) is that determined by Forneris et al. (2007) in complex with substrate-like peptide inhibitor. The three-dimensional structure of Arabidopsis LDL3, as well as of Physcomitrella and Selaginella LDL3 homologs (AtLDL3, PpLDL3, SmLDL3, respectively) were obtained through molecular modeling approaches based on the three-dimensional structure of HsLDS1 and the molecular model of Arabidopsis LDL1 (Spedaletti et al., 2008). The FAD cofactor is shown in ball-and-stick representation and colored in blue. Shaded ellipses highlight the ‘Tower’ domain of HsLSD1 and PpLDL3, and the corresponding regions in SmLDL3 and AtLDL3.

LDL1, LDL2, and FLD do not have the HsLSD1 protruding ‘Tower’ domain. However, despite the absence of the ‘Tower’ domain, demethylase activity has been shown for Arabidopsis LDL1 (Spedaletti et al., 2008), as shown for the mouse LSD2 which also lacks the ‘Tower’ domain (Karytinos et al., 2009). Conversely to LDL1, LDL2, and FLD, a small insertion (about 33 amino acids) is present inside the AO domain of LDL3 (Figure 1), which, however, has low sequence similarity to the HsLSD1 ‘Tower’ domain. Molecular modeling of LDL3 indicates that this region is probably unstructured (Figure 2). Nonetheless, it cannot be excluded that this region becomes structured upon interaction with, yet unknown, binding partners. Furthermore, comparative analysis of the LDL3 model with respect to the three-dimensional structure of HsLDS1 in complex with a substrate-mimic peptide (Forneris et al., 2007) and to the molecular model of LDL1 (Spedaletti et al., 2008) indicates that almost all of the residues involved in substrate binding in HsLSD1 are conserved in both LDL1, whose demethylase activity has been demonstrated experimentally (Spedaletti et al., 2008), and LDL3 (Table 1). This analysis suggests that LDL3 is a lysine demethylase with a substrate specificity similar to that of LDL1.

**Table 1:** Substrate binding residues in human LSD1, and orthologous residues in Arabidopsis LDL1 and LDL3.

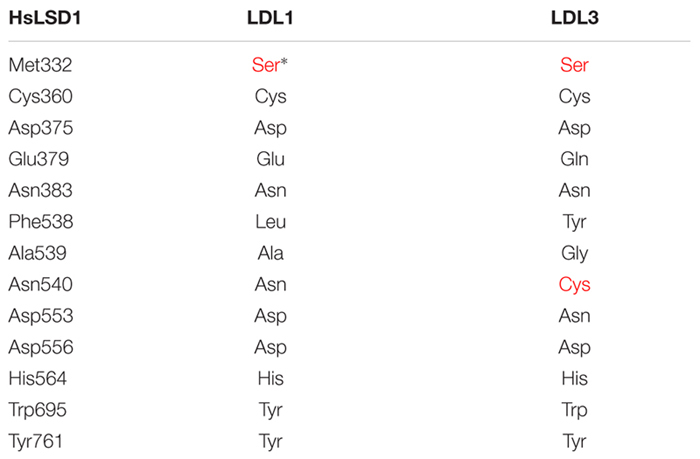

*Binding residues have been selected on the basis of the three-dimensional structure of human LSD1 (HsLSD1) in complex with a substrate-mimic peptide (PDB code 2V1D; Forneris et al., 2007). Orthologous residues in Arabidopsis LDL1 and LDL3 have been identified on the basis of structure-based sequence alignment using the molecular models of Arabidopsis LDL1 (Spedaletti et al., 2008) and LDL3 (present work). ^∗^Conserved or conservatively substituted residues are shown in black, non-conserved residues in red.*

### Evolutionary History of Plant LDL/FLDs

**FIGURE 3:**
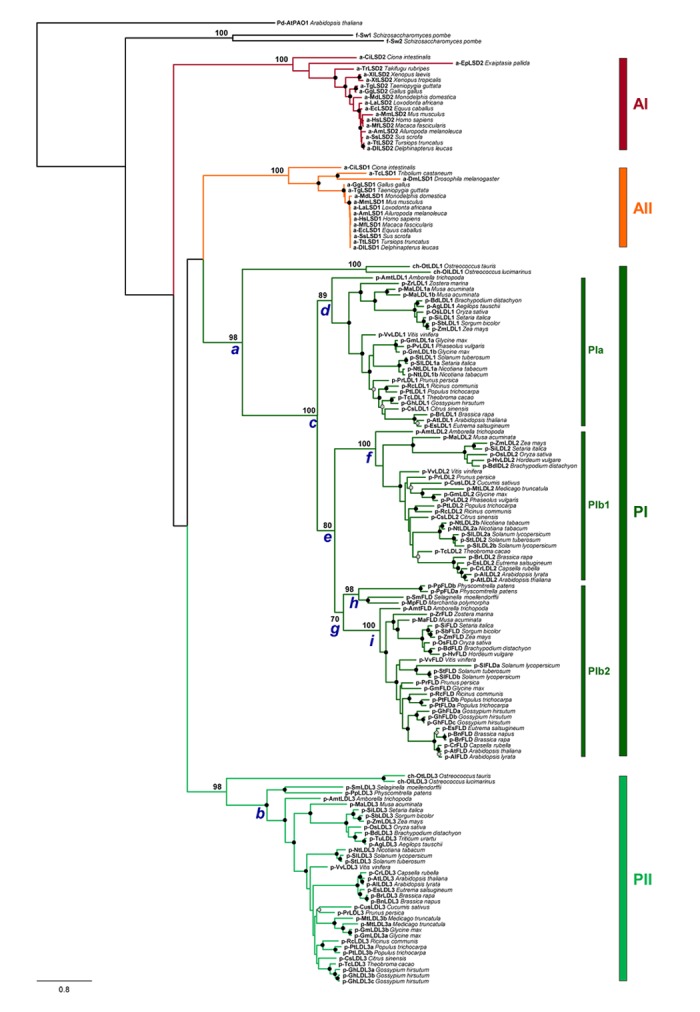
Phylogenetic tree of full-length amino acid sequence of LDL/FLD homologs in representative plant species. Animal HsLSD1 and HsLSD2 homologs, as well as the two *Schizosaccharomyces pombe* homologs SWIRM1 and SWIRM2 (Nicolas et al., 2006) are also included in this analysis. Phylogenetic analyses were performed with the Maximum Likelihood method using RAXML v.8.2.10 (Stamatakis, 2014) with the PROTGAMMAJTT substitution model. Node support was evaluated with 1,000 rapid bootstrap inferences. The sequence of the polyamine oxidase 1 of *A. thaliana* (AtPAO1; At5g13700; Supplementary Table 1) was used as outgroup.

To investigate the evolutionary history of the plant LDL/FLDs, a phylogenetic analysis of the amino acid sequence of 159 LDL/FLD homologs from 57 different representative animal, plant and algal species (Supplementary Table 1) was carried out. Maximum likelihood phylogenetic trees of both the full-length (Figure 3) and AO domain (Supplementary Figure 1) amino acid sequences show four main clades: clades AI and AII grouping, respectively, HsLSD1 and HsLSD2 homologs from animals, and clades PI and PII grouping LDL/FLD homologs from plants and algae. Relationships between these animal and plant clades are unclear since the most basal nodes of the tree lack strong bootstrap support, in agreement with a recent phylogenetic analysis of the AO domains based on a limited number of plant and animal LDL/FLDs (Zhou and Ma, 2008). Within both clade PI and clade PII, green algae LDL/FLDs form a subclade that is sister to the clade formed by land plant LDL/FLDs (Figure 3 and Supplementary Figure 1), in agreement with organism relationships. Phylogenetic relationships between plant LDL/FLDs indicate that plant LDL1, LDL2, and FLD homologs share a recent common ancestor (Figure 3, node *c*; bootstrap support, DBS = 100), whereas plant LDL3 homologs belong to a different evolutionary lineage (node *b*; BS = 98), consistently to what has been suggested for the Arabidopsis LDL/FLDs based on gene structure analysis. Moreover, within clade PI, plant LDL1, LDL2, and FLD homologs form three well supported clades (clades PIa, PIb1, and PIb2, respectively), with LDL2 homologs sister to FLD homologs (node *e*; BS = 80). The LDL/FLD homologs of PIa, PIb1, and PIb2 clades are well distributed among the various flowering plant species, being present both in dicotyledonous and monocotyledonous plants, as well as in *Amborella trichopoda*, which represents the sister lineage to all other extant flowering plants (Amborella Genome Project, 2013). In these clades, phylogenetic relationships between LDL/FLD amino acid sequences closely reflect evolutionary relationships between plant families to which they belong (Figure 3). This phylogenetic pattern suggests that *LDL1*, *LDL2*, and *FLD* genes have evolved through gene duplications. A first duplication would have occurred in correspondence of node *c* and a second one at node *e*. The fact that *Amborella trichopoda* shows one copy of each of *LDL1*, *LDL2*, and *FLD* genes with sister relationships to the corresponding clades formed by flowering plant LDL/FLD homologs indicates that such duplication events occurred before the split between eudicots and monocots. Most likely duplications took place early during the diversification of land plants, as suggested by the occurrence of a supported subclade of FLD (Figure 3, node *h*; BS = 98) clustering homologs found in the moss *Physcomitrella patens*, the liverwort *Marchantia polymorpha* and the seedless ancient vascular plant *Selaginella moellendorffii*. According to this scenario, gene duplications would have been followed by *LDL1* and *LDL2* gene loss in these ancient plants. Phylogenetic relationships between plant LDL1, LDL2, and FLD homologs also account for their shared gene structure. Indeed, with some exceptions, in most flowering plants *LDL1* homologs lack introns, *LDL2* homologs display one intron, whereas *FLD* homologs have four to five introns, all at conserved positions (Table 2 and Supplementary Table 2). This suggests that sequential insertions have occurred first in the common ancestor of *LDL2* and *FLD* genes (node *e*) and later in the ancestor of *FLD* genes (node *g*).

**Table 2:** Characteristics of LSD1-like proteins in various organisms.

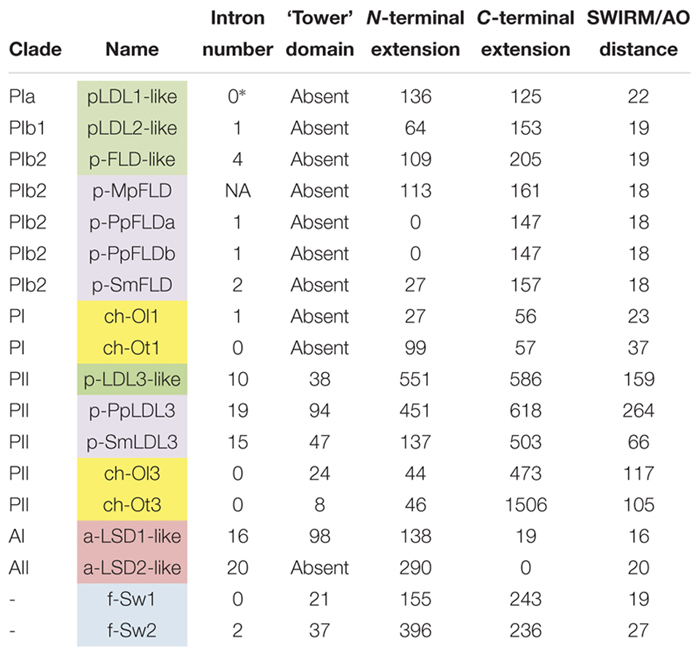

*The number of introns in the corresponding genes and the number of the amino acid residues constituting the ‘Tower’ domain, the *N*- and *C*-terminal extensions and the distance between the SWIRM and amine oxidase (AO) domains are indicated. ^∗^For p-LDL1-, p-LDL2-, p-FLD-, p-LDL3-, a-LSD1-, and a-LSD2-like proteins, mean values from all the flowering plant (p-) and animal (a-) species considered in the present study (Supplementary Figure 2) are indicated. Complete data are reported in Supplementary Figure 2. Pp, *Physcomitrella patens*; Sm, *Selaginella moellendorffii*; Mp, *Marchantia polymorpha*; Ol, *Ostreococcus lucimarinus*; Ot, *Ostreococcus tauri*; f-Sw1 and f-Sw2, LSD1-like proteins from *Schizosaccharomyces pombe*. NA, genomic sequence not available.*

The phylogenetic pattern observed in lineage PII provides no evidence for old duplication events behind the diversification of LDL3 homologs, whereas species-specific duplication events might account for the occurrence of multiple *LDL3* genes in the rosids *Gossypium hirsutum*, *Populus trichocarpa*, and *Glycine max.* Furthermore, the *LDL*/*FLD* homologs of group PII bear a higher number of introns (5 to 19 introns, Table 2 and Supplementary Table 2) than those of clade PI. Whether such difference among *LDL*/*FLD*s of clade PI and clade PII has a physiological significance is still unknown. Also animal *LSD1* and *LSD2* homologs have a large number of introns (larger than the plant *FLD* and *LDL3* homologs; Table 2), not at conserved positions when the genes of the two animal clades are compared. These data suggest a different evolutionary history for the two animal clades.

Phylogenetic results can also provide important insight into the structural evolution of LDL/FLDs and LSDs. Several structural differences can be pointed out among the different plant and animal clades. In particular, all LDL/FLD homologs of group PII have long *N*- and *C*-terminal extensions, as well as long SWIRM/AO regions, as compared the LDL/FLD homologs of group PI, with the exception of the LDL3 homologs of the two green algal species and *S. moellendorffii* which display short *N*-terminal extensions (Table 2). Also animal LSD homologs of both clade AI and AII have large *N*-terminal extensions, but small or null *C*-terminal extensions (Table 2). In particular, LSD2 homologs display longer *N*-extensions with respect to the LSD1 homologs, probably through acquisition of DNA- or protein-interaction domains. Indeed, HsLSD2 possesses both CW-type and C4H2C2-type zinc finger motifs (Burg et al., 2015). Furthermore, similarly to the Arabidopsis LDL3, all LDL/FLD homologs of group PII, except the *Ostreococcus tauri* one, are characterized by the presence of a small insertion inside the AO domain (Table 2), at the same position of the ‘Tower’ domain in the animal LSD1 homologs. This insertion is smaller than the HsLSD1 ‘Tower’ domain, being of 47 amino acids in *S. moellendorffii*, 50 amino acids in *A. trichopoda*, 39 to 43 amino acids in monocots, 33 to 39 amino acids in dicots, and 24 amino acids in *Ostreococcus lucimarinus*. Only the LDL3 homolog of *P. patens* (PpLDL3) displays an insertion of a size (94 amino acids) similar to that of the animal ‘Tower’ domain (Table 2). Molecular modeling analyses indicate that this insertion may adopt a fold similar to that of the HsLSD1 ‘Tower’ domain (Figure 2). In contrast, the *S. moellendorffii* insertion appears unstructured (Figure 2). The functional significance of these structural differences among the different plant and animal LDL/FLDs and LSDs are not clear so far.

### Expression Pattern of the Four Arabidopsis *LDL*/*FLD* Genes During Seedling Development

**FIGURE 4:**
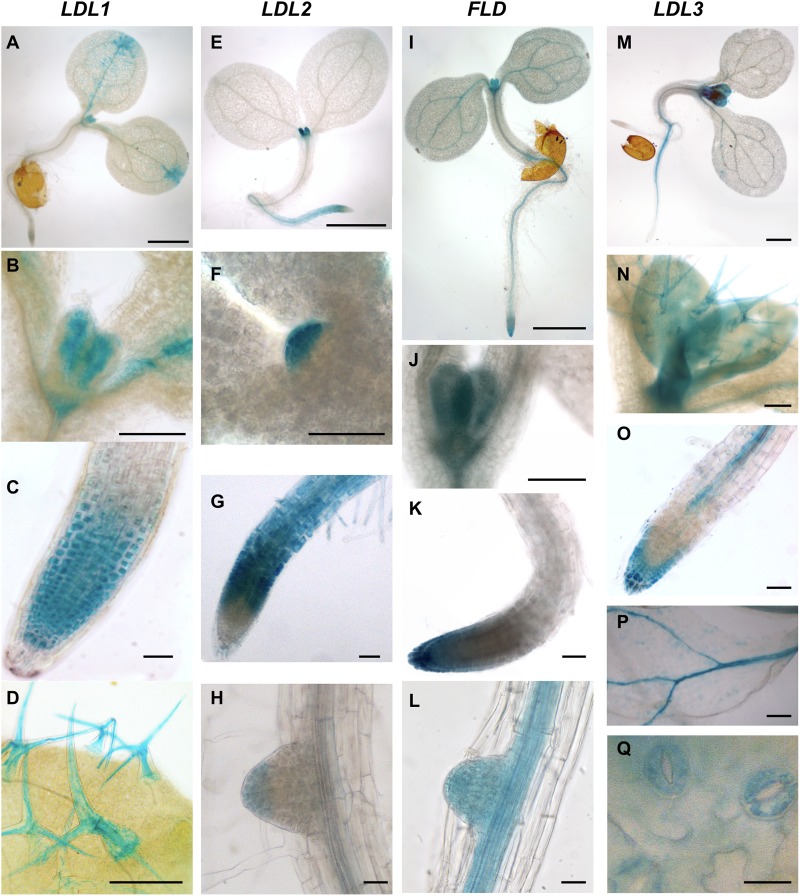
Promoter activity of Arabidopsis *LDL*/*FLD* genes during vegetative development. Histochemical GUS staining of *LDL/FLD::GFP-GUS* Arabidopsis seedlings is shown. Bars indicate 1 mm in **(A,E,I,M)**, 200 μm in **(N)**, 100 μm in **(B–D,F–H,J–L,O,P)**, and 20 μm in **(Q)**.

Since information concerning the tissue- and organ-specific gene expression pattern may be useful to determine physiological roles, promoter regions of the four Arabidopsis *LDL*/*FLD* genes were cloned upstream of a GFP-GUS fusion gene, and *LDL/FLD::GFP-GUS* transgenic Arabidopsis plants were obtained. Histochemical GUS staining of developing seedlings showed that *LDL1* is expressed in the shoot apical meristem (SAM), in the newly emerging leaves (Figure 4A,B), and in the root tip (Figure 4C). Cotyledons also appeared stained mainly at the tips and along the vascular system (Figure 4A). Strong *LDL1*-specific GUS staining was also observed in trichomes (Figure 4D). *LDL2*-specific GUS staining was observed in the root elongation and differentiation zones up to the meristematic region and in the columella of primary and secondary roots (Figure 4E,G,H). *LDL2* expression was also observed in the SAM and the newly emerging leaves (Figure 4E,F). *FLD* is expressed in the root apex of primary (Figure 4I,K) and secondary roots (Figure 4L), in the SAM and in the newly emerging leaves (Figure 4I,J). It is also expressed in the vascular system of cotyledons, roots (Figure 4I,L) and fully developed leaves. *LDL3* is expressed in newly emerging leaves (Figure 4M,N), in the columella and in the root vascular system (Figure 4M,O). *LDL3*-related GUS staining was also observed in the vascular system of leaves (Figure 4P), in guard cells (Figure 4Q), and in trichomes (Figure 4N).

### Expression of Arabidopsis *LDL*/*FLD* Genes During Flower Development and Embryogenesis

**FIGURE 5:**
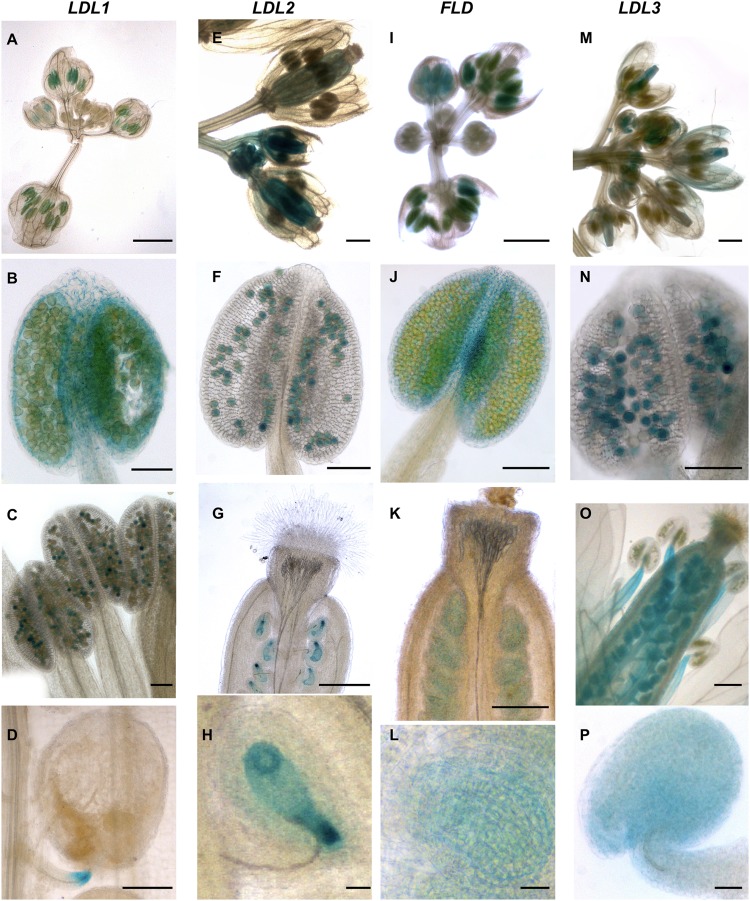
Promoter activity of Arabidopsis *LDL*/*FLD* genes during reproductive development. Histochemical GUS staining of *LDL/FLD::GFP-GUS* Arabidopsis transgenic plants in inflorescences is shown. Bars indicate 1 mm in **(A,E,G,I,K,M,O)**, 100 μm in **(B–D,F,J,N)**, and 20 μm in **(H,L,P)**.

**FIGURE 6:**
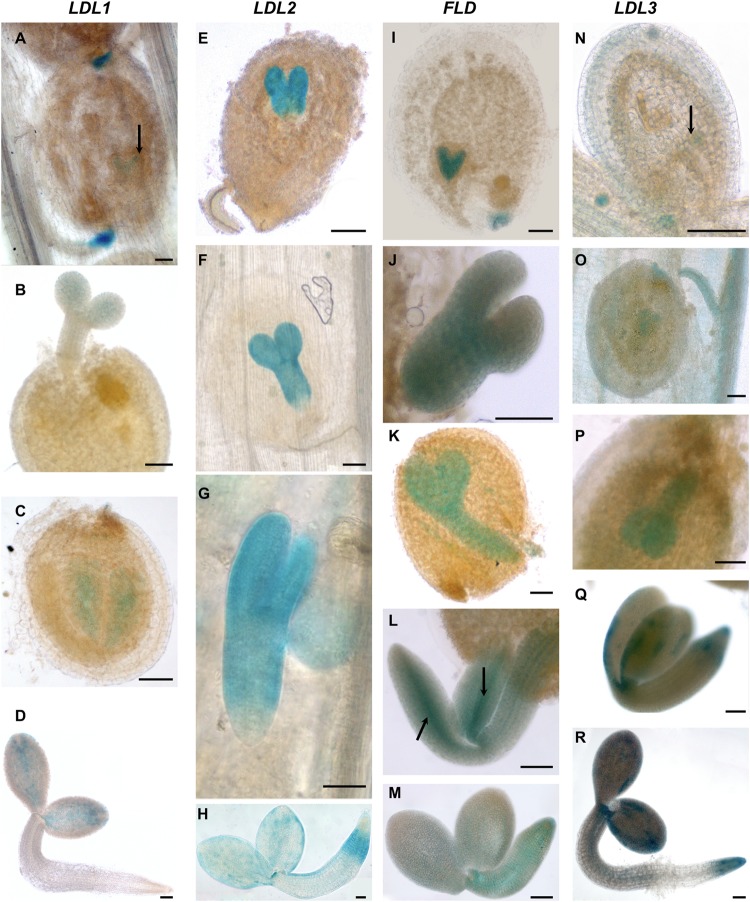
Promoter activity of Arabidopsis *LDL/FLD* genes during embryogenesis and in imbibed seeds. Histochemical GUS staining of *LDL/FLD::GFP-GUS* transgenic plants in siliques **(A–C,E–G,I–L,N–Q)** and imbibed seeds **(D,H,M,R)**. Black arrows indicate *LDL1*-specific staining in embryo **(A)**, *FLD*-specific staining in provascular tissues of roots and cotyledons in embryo **(L)** and *LDL3*-specific staining in globular stage of embryo **(N)**. Bars indicate 50 μm.

*LDL1*-related GUS signal was observed in young, completely closed floral buds (Figure 5A). Staining in developing anthers and in particular in both anther tapetum and filaments was also observed (Figure 5B). In later steps of flower development, strong GUS staining was evident in mature pollen grains (Figure 5C), while in non-fertilized ovules only faint staining was observed. During embryo development, *LDL1*-related GUS staining was present in the funiculus of the fertilized ovule, mainly at the ovule proximal region (Figure 5D). Furthermore, developing and mature embryos presented staining at the central part of cotyledons (Figure 6A–C) and this pattern was maintained in fully developed embryos, as observed by GUS histochemical analysis of imbibed seeds (Figure 6D). The *LDL1* expression in imbibed seeds is in agreement with the public Arabidopsis microarray database and the reported essential role of *LDL1* and *LDL2* in seed dormancy (Zhao et al., 2015).

*LDL2*-specific GUS staining was observed in developing pistils and anthers of floral buds (Figure 5E). Later during development, mature pollen grains (Figure 5F) and embryo sacs (Figure 5G,H) were stained too, embryo sacs presenting a strong signal at the micropylar end (Figure 5H). Following fertilization, strong *LDL2*-specific staining was observed in developing embryos at the heart and torpedo stages and in mature embryos (Figure 6E–G). This staining was extended in the entire embryo, excluding only the embryonal root tip. In embryos within the imbibed seeds, the expression pattern resembled the one of the young seedlings, with strong promoter activity in SAM, cotyledons, and in the root elongation zone (Figure 6H).

*FLD-*related GUS staining was observed in the anther–filament junction and in the tapetum (Figure 5I,J), but not in mature pollen grains. Ovules also appeared stained (Figure 5K,L). Following fertilization, strong *FLD*-specific staining was observed in developing embryos at the heart and torpedo stages and in mature embryos (Figure 6I–L). Mature embryos in imbibed seeds also presented staining (Figure 6M). Interestingly, *FLD* expression was evident in the provascular tissues of embryonic roots and cotyledons (Figure 6L, arrows), similarly to *FLD* expression in root, cotyledon and leaf vascular system of young seedlings (Figure 4I,L).

*LDL3*-specific staining was observed in peduncles, sepals (Figure 5M), stamen filaments (Figure 5O) and mature pollen grains (Figure 5N). Pistils were also stained (Figure 5M,O), in particular ovules (Figure 5O,P). Following fertilization, *LDL3*-specific staining was observed both in developing and in mature embryos (Figure 6N–Q). Funiculus of fertilized ovules presented staining as well (Figure 6O). In mature embryos, staining of the SAM and root tip was evident (Figure 6Q). The same staining pattern was observed in mature embryos inside imbibed seeds (Figure 6R).

### *LDL3* Mutant Plants Show Early-Flowering Phenotype

**FIGURE 7:**
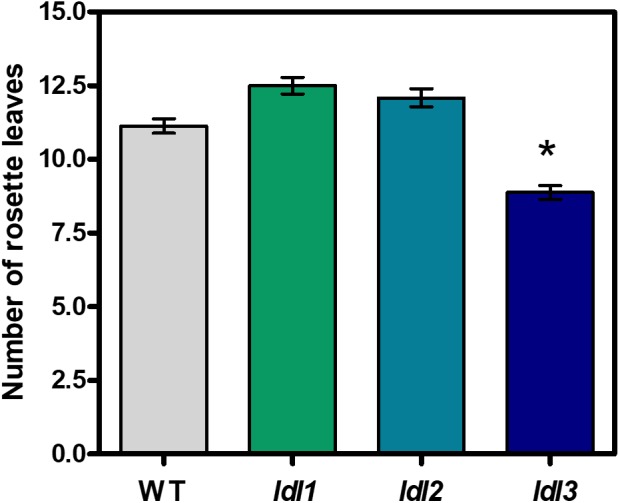
Flowering time of *ldl*/*fld* loss-of-function Arabidopsis mutants for *LDL*/*FLDs*. Flowering time of *ldl1*, *ldl2*, *ldl3* mutants and wild-type plants (WT) is expressed as the number of rosette leaves at bolting. The *fld* mutant plants presented non-flowering phenotype under our growth conditions. Mean values (*n* > 8) of a representative analysis out of at least three repetitions are shown and bars indicate standard error. Asterisk indicates statistically significant differences from WT plants (one-way ANOVA test, *p* < 0.05).

To elucidate the physiological roles of the four Arabidopsis *LDL*/*FLD* genes, loss-of-function mutants for each of the four Arabidopsis *LDL*/*FLD* genes were identified and characterized to confirm disruption of gene expression (Supplementary Figure 2). The *ldl*/*fld* mutants were initially examined for flowering time by measuring the number of rosette leaves upon bolting. In agreement with previously published data (He et al., 2003), the *fld* mutant presented an extremely late-flowering phenotype. Indeed, floral transition was not obtained under our experimental conditions unless the *fld* mutant plants were treated with gibberellins or grown under low-temperature conditions for prolonged periods, further confirming that FLD is involved in the autonomous pathway controlling flowering time. Under the same conditions, the *ldl1* and *ldl2* mutants displayed only a very short, not statistically significant, delay in flowering (Figure 7) as previously reported (Jiang et al., 2007). Conversely, data presented here evidence that *ldl3* mutant display early-flowering phenotype (Figure 7).

### *FLC* Is Up-Regulated in *ldl1*, *ldl2*, and *fld* Mutants, but Down-Regulated in *ldl3* Mutants

**FIGURE 8:**
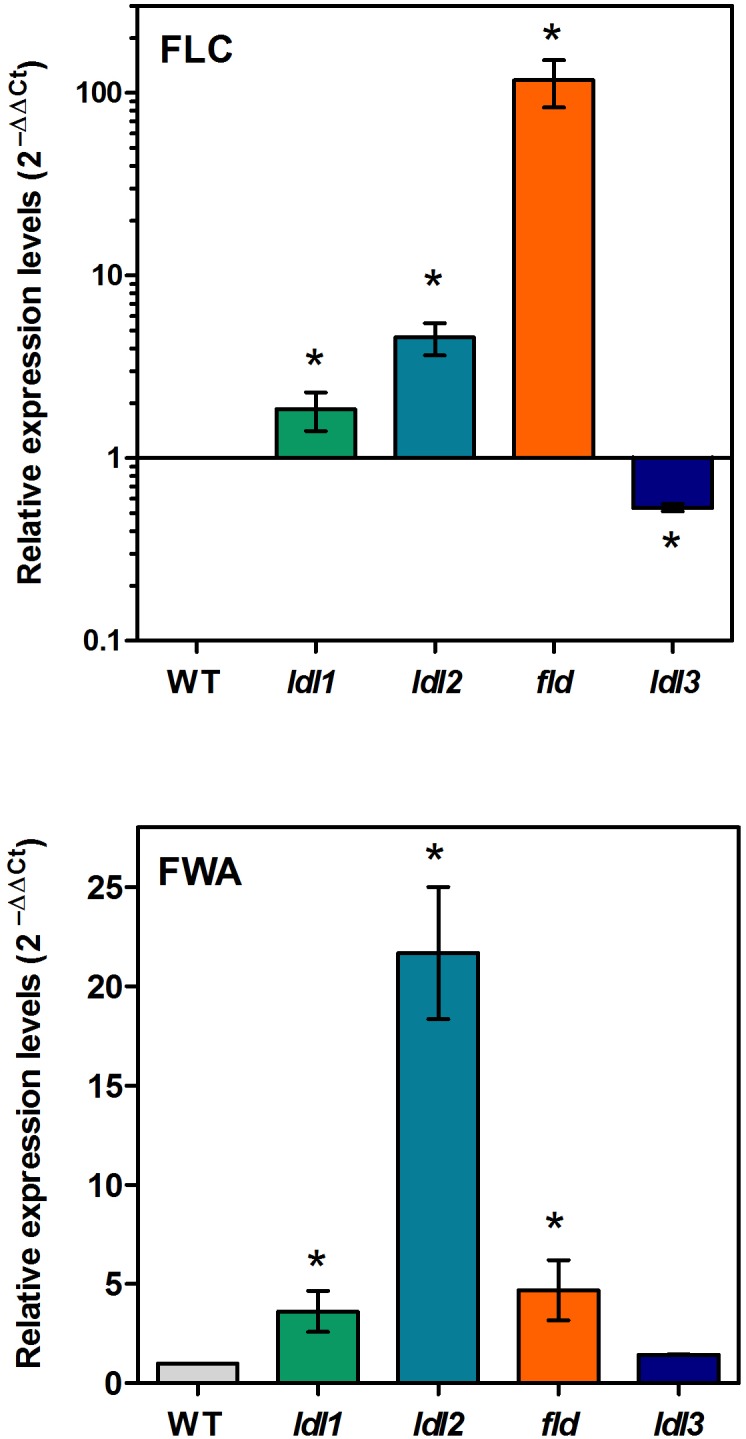
Relative expression levels of *FLC* and *FWA* in Arabidopsis mutants for *LDL*/*FLDs*. Two-week-old seedlings of *ldl1*, *ldl2*, *fld*, and *ldl3* mutants, and wild-type plants (WT) were analyzed for *FLC* and *FWA* expression levels by qRT-PCR. Data for *FLC* are shown in logarithmic scale. Numbers are mean values ± SE of three independent replicates. Asterisks indicate statistically significant differences from WT plants (one-way ANOVA test, *p* < 0.05).

To verify whether the early-flowering phenotype of the *ldl3* mutant depends on *FLC*, as the late-flowering phenotype of *ldl1, ldl2*, and *fld* mutants does (He et al., 2003; Jiang et al., 2007), a comparative analysis of the *FLC* expression levels in the four *lsd/fld* mutants was performed. The qRT-PCR analysis evidenced twofold and fourfold increase in *FLC* expression levels in the *ldl1* and *ldl2* mutants, respectively, comparing to the wild-type plants, as opposed to a 100-fold increase in the *fld* mutant (Figure 8), consistently with the flowering phenotypes. Conversely, a twofold decrease in *FLC* expression levels was observed in the *ldl3* mutant as compared to the wild-type plants (Figure 8), which is also consistent with the early-lowering phenotype of this mutant. These results suggest that the various members of *LDL*/*FLD* gene family contribute in a different way to the control of *FLC* expression and thus to the flowering time, *LDL3* having an opposing effect with respect to the others.

MAF1 to MAF5, MADS-containing transcription factors homologs to FLC, also contribute to the control of the flowering time (Ratcliffe et al., 2003; Rosloski et al., 2013). Data from qRT-PCR analysis evidenced no statistically significant difference in the expression levels of *MAF1* to *MAF4* in all four *ldl*/*fld* mutants as compared to the wild-type plants (Supplementary Figure 3). These data indicate that Arabidopsis *LDL/FLDs* are not involved in the regulation of the *MAF* gene family despite the fact that *fld* mutants were previously shown to display altered H3K4 trimethylation levels at *MAF4* and *MAF5* (Yu et al., 2011).

FWA is a transcription factor which participates in the control of floral transition and which has been demonstrated to be under epigenetic control. In particular, *FWA* is silenced during plant vegetative development and in the sporophytes by repressive DNA methylation in its 5′ region, its expression being confined to the central cell of the female gametophytes and to the endosperm. Moreover, *fwa* epi-alleles cause a late-flowering phenotype due to ectopic *FWA* expression in sporophytic tissues (Soppe et al., 2000; Kinoshita et al., 2004). In addition, *FWA* was shown to be ectopically activated in rosette leaves of *ldl1* and *ldl2* mutants, but not of *fld* mutants, suggesting that LDL1 and LDL2 contribute to the repression of *FWA* expression during vegetative development (Jiang et al., 2007). Here, to determine the specific contribution of the four LDL/FLDs to *FWA* regulation, qRT-PCR analysis was performed. Results showed a strong increase in *FWA* expression levels in the *ldl2* mutant and a smaller one in the *ldl1* and *fld* mutants (Figure 8). Instead, no difference in *FWA* expression levels was observed in the *ldl3* mutant as compared to the wild type plants. These data suggest that it is mainly LDL2, among the four Arabidopsis LDL/FLDs, that is involved in *FWA* repression during vegetative growth of Arabidopsis plants. This well correlates with the *LDL2* expression in the female gametophyte presenting a pattern similar to that of *FWA* (Kinoshita et al., 2004). Indeed, both *LDL2* and *FWA* are expressed in embryo sacs (Figure 5N) (Kinoshita et al., 2004).

## Discussion

In the present work, a comparative study on gene structure, phylogenetic relationships, spatio-temporal expression patterns, physiological roles and target genes for the four *LDL*/*FLD* genes was performed, which evidenced several similarities, but also important differences among them.

Data from exon/intron structure analyses and phylogenetic studies suggest that the *LDL1*, *LDL2*, and *FLD* homologs of the various plant species derive from a single copy of the *LDL*/*FLD* gene present in the early ancestor of land plants through two duplication events. Furthermore, the different number of introns observed in *LDL*/*FLD* genes is likely the result of two sequential insertion events occurred first in the common ancestor of *LDL2* and *FLD* genes and later in the ancestor of *FLD* genes. This process might have brought about differences in expression levels and function among *LDL*/*FLD* homologs of clade PI. Indeed, it has been shown that although all three LDL1, LDL2, and FLD act redundantly in the control of the flowering time, FLD plays a major role in this process (Jiang et al., 2007). Intron acquisition occurred also during evolution of the *LDL3* homologs which might have contributed to increase gene expression levels. Differently from LDL1, LDL2, and FLD, LDL3 homologs are characterized by the presence of a small insertion in the same position as that of the ‘Tower’ domain in HsLSD1. This insertion, which in *P. patens* is long enough to allow the presence of a structural domain similar to the HsLSD1 ‘Tower’ domain, became shorter during the transition from the moss *P. patens* (94 amino acids), to the ancient vascular plant *S. moellendorffii* (47 amino acids), to the basal angiosperm *A. trichopoda* (50 amino acids), to dicots and monocots (33–47 amino acids). The evolutionary pressure leading to such changes is not known yet. It is possible that these changes have been accompanied by evolution of new protein/protein interaction motifs. Altogether, these data indicate a different evolutionary history of the two main plant *LDL*/*FLD* clades, similarly to the two animal *LSD* clades.

In the present study, an analysis of the main LDL/FLD target genes showed that all four LDL/FLDs are involved in the control of *FLC* expression. In particular, in agreement with previously published data (Jiang et al., 2007), LDL1, LDL2, and FLD were shown to have a repressive effect on *FLC* expression levels, with the effect of FLD being much more pronounced than that of LDL1 and LDL2. Instead, LDL3 has an enhancing effect on *FLC* expression. Indeed, *ldl3* mutant plants display decreased *FLC* transcript levels, as compared to the wild-type plants, while *ldl1*, *ldl2*, and *fld* mutants display increased *FLC* levels (Figure 8). These differences in *FLC* expression levels reflect the differences in flowering time, *fld* displaying a non-flowering phenotype, while *ldl3* an early-flowering phenotype (Figure 7). LDL1, LDL2, and FLD repress also *FWA* expression LDL2 having a more pronounced effect than LDL1 and FLD (Figure 8). The lack of differences in flowering time among *ldl1* and *ldl2* mutants and wild-type plants, despite the altered *FLC* and *FWA* expression levels, suggests a quantitative effect of *FLC* and *FWA* on floral transition. Differences in tissue- and temporal-specific expression pattern, as well as in protein/protein interactions among the four *LDL*/*FLDs* may also explain the different flowering phenotypes of the four *ldl*/*fld* mutants.

Previous studies have shown that LDL1, LDL2, and FLD repress *FLC* transcription by reducing H3K4 methylation levels at specific regions of the *FLC* chromatin (Jiang et al., 2007). This raises the question of which are the underlying mechanisms determining the opposing effects of the different LDL/FLDs on *FLC* expression levels considering the similarity of the catalytic sites (Table 1) (Spedaletti et al., 2008). To get through these mechanisms, the LDL3 substrate specificity, the *FLC* chromatin regions with which LDL3 specifically interacts and the LDL/FLD specific partners have to be determined. On the other hand, the physiological significance of the different/opposing effects of the four LDL/FLDs on floral transition is not clear so far. They may contribute to a fine-tune regulation and optimization of the flowering time.

*FLC* expression is promoted by FRIGIDA (FRI) and is repressed by sets of genes in the autonomous and vernalization pathways (Amasino and Michaels, 2010; Yang et al., 2017). *FLC* is expressed in shoot and root apical regions, as well as in leaf vasculature, in pollen mother cells, in the tapetum surrounding these cells, and in the anther connective tissue, but not in mature pollen grains (Bastow et al., 2004; Michaels et al., 2005; Sheldon et al., 2008; Choi et al., 2009). It is also expressed in the ovule integuments before and after pollination, but not in the female gametophytes. *FLC* is additionally expressed in the developing embryo during all stages of embryogenesis reaching a maximum when the seed has been fully formed (Sheldon et al., 2008; Choi et al., 2009; Berry and Dean, 2015). In old embryos, *FLC* is expressed in the provascular tissue of both the embryonic roots and cotyledons. Thus, *FLC* expression is repressed in mature male and female gametophytes to be reactivated after fertilization, in reprogramming processes that are considered important for plant reproduction, mainly ensuring a vernalization requirement in each generation (Berry and Dean, 2015). Little is known so far about how the several *FLC* regulators control *FLC* transcription in the various developmental stages (Choi et al., 2009). The promoter activity studies presented here evidence that all four Arabidopsis *LDL*/*FLD* genes are expressed in SAM and/or newly emerging leaves. The Arabidopsis *LDL*/*FLD* genes are also expressed in roots, though with a gene-specific pattern. Furthermore, all *LDL*/*FLD* genes, except *LDL*2, are expressed in the vascular system of the roots and/or leaves. *LDL3*, differently from the other *LDL*/*FLD* genes, is also expressed in guard cells. *LDL*/*FLDs* are also expressed during reproductive development, though with some differences from each other. In particular, prior to fertilization all four *LDL*/*FLDs* are expressed in ovules. However, while *LDL1*, *FLD* and *LDL3* are expressed in the entire ovule, probably mainly involving the ovule integuments, *LDL2* is specifically expressed in the embryo sacs. Furthermore, *LDL1*, *LDL2*, and *LDL3* are expressed in mature pollen grains, as opposed to *FLD* that is not expressed in male gametophytes. Following fertilization, all four *LDL*/*FLDs* are expressed in developing embryos. *LDL1* and *LDL3* are additionally expressed in the funiculus of developing embryos. Altogether, the promoter activity studies presented here show that the four *LDL*/*FLDs*, both the *FLC* repressors and the FLC activator, display overlapping and complementary expression patterns with respect to each other and to *FLC*, thus not allowing to assign a specific role to each of them in *FLC* regulation at certain developmental stages. What appears to be an important difference among the different *LDL*/*FLDs* is the lack of *FLD*-specific expression in pollen grains. These data exclude the possibility that FLD, which among the four LDL/FLDs is the best *FLC* repressor, is responsible for *FLC* repression in pollen grains, in agreement with previous data showing that *FLC* expression pattern during gametogenesis and embryogenesis is not altered in an *fld* genotype (Choi et al., 2009). LDL1 and LDL2 may have a role in this process, although it is again difficult to explain the presence of LDL3, which acts as an FLC activator, in pollen. Further detailed analyses of *FLC* expression pattern in single and multiple *ldl*/*fld* mutants may give useful information on the specific contribution of the different *LDL*/*FLDs* to *FLC* regulation in a tissue- and organ-specific way and in the reprogramming processes. It is also likely that a balanced activity of different FLC regulators is necessary for proper *FLC* levels to be established at the various developmental stages. On the other hand, the high expression levels of *LDL*/*FLDs* during gametogenesis and embryogenesis suggests a function for this gene family in the transgenerational reset of epigenetic memory, known to affect not only DNA methylation level, but also histone methylation (Zheng et al., 2016).

*FWA* is specifically expressed in the female gametophytes, mainly in the central cell and in the developing endosperm, for 48 h after pollination (Kinoshita et al., 2004). However, in the present study, a similar expression pattern has been evidenced for LDL2 (Figure 5H), which, among the three LDL/FLDs, appears to have a major role in the control *FWA* expression during vegetative growth of Arabidopsis plants, repressing it. LDL2 may be necessary together with FWA activators in multi-protein complexes for optimal *FWA* levels in embryo sacs. It is also possible that LDL2 is responsible for repression of *FWA* expression in ovules 48 h after pollination (Kinoshita et al., 2004).

Altogether, data presented here suggest functional differences among the four Arabidopsis *LDL*/*FLD* genes, even among the *LDL1*, *LDL2*, and *FLD*, which are recent derivatives of a common ancestor gene. It is possible that following gene duplication, *LDL1*, *LDL2*, and *FLD* genes have undergone sub-functionalization or neo-functionalization which might have helped in the optimization of the regulatory network controlling floral transition and defense responses (Zhou and Ma, 2008).

Several studies have evidenced a relevant role of the different epigenetic mechanisms in the control of plant developmental and defense/adaptation processes and their impact on agronomical traits other than flowering time, such as yield and fruit ripening (Gallusci et al., 2017; Giovannoni et al., 2017; Annacondia et al., 2018). In this context, the contribution of the *LDL*/*FLD* gene family in these processes should be analyzed and involved target genes should be identified. These pieces of information may provide novel biotechnological strategies for crop improvement.

## Author Contributions

PT conceived the research plan. DM and PT designed and performed the experiments. BB, DS, and PT contributed to the phylogenetic analyses. FP performed the molecular modeling analyses. DM, DS, FP, and PT wrote the manuscript. AC and RA provided advice and comments for the manuscript.

## Conflict of Interest Statement

The authors declare that the research was conducted in the absence of any commercial or financial relationships that could be construed as a potential conflict of interest.

## Footnotes

1 https://github.com/ddarriba/modeltest

2 http://www.phylo.org/
