# Supplementary material for: The Four FAD-Dependent Histone Demethylases of Arabidopsis Are Differently Involved in the Control of Flowering Time
Source: Front Plant Sci. 2019 Jun 4;10:669. doi: 10.3389/fpls.2019.00669 (PMC6558185; doi:10.3389/fpls.2019.00669)
Supplement: Supplementary file 1 [file Table_1.pdf]

Supplementary Table 1. FAD-dependent lysine-specific histone demethylases used in phylogenetic analyses (NA:genomic sequences not available).

Clade	Sequence code	Organism	Accession number	Number of introns	*N*-extension	SWIRM/AO distance	Tower domain	*C*-extension
AI	a-AmLSD2	*Ailuropoda melanoleuca*	XP011226672.1	21	275	20	absent	0
AI	a-CiLSD2	*Ciona intestinalis*	XP002121982.1	22	500	25	absent	9
AI	a-DlLSD2	*Delphinapterus leucas*	XP022442447.1	23	277	20	absent	0
AI	a-EcLSD2	*Equus caballus*	XP005603653.1	21	277	20	absent	0
AI	a-EpLSD2	*Exaiptasia pallida*	KXJ14910.1	15	242	16	absent	193
AI	a-GgLSD2	*Gallus gallus*	XP015131437.1	20	277	20	absent	0
AI	a-HsLSD2	*Homo sapiens*	XP005248983.1	22	279	20	absent	0
AI	a-LaLSD2	*Loxodonta africana*	XP003416501.1	24	277	20	absent	0
AI	a-MfLSD2	*Macaca fascicularis*	XP005554000.1	21	279	20	absent	0
AI	a-MdLSD2	*Monodelphis domestica*	XP007487955.1	21	279	20	absent	0
AI	a-MmLSD2	*Mus musculus*	NP758466.1	20	285	22	absent	0
AI	a-SsLSD2	*Sus scrofa*	XP001927879.1	21	277	20	absent	0
AI	a-TgLSD2	*Taeniopygia guttata*	XP002190331.1	19	277	20	absent	0
AI	a-TrLSD2	*Takifugu rubripes*	XP011618591.1	20	294	18	absent	0
AI	a-TtLSD2	*Tursiops truncatus*	XP019800622.1	23	277	20	absent	0
AI	a-XlLSD2	*Xenopus laevis*	XP018123297.1	20	278	20	absent	0
AI	a-XtLSD2	*Xenopus tropicalis*	XP002932724.1	21	278	20	absent	0
AII	a-AmLSD1	*Ailuropoda melanoleuca*	XP019655525.1	20	116	16	97	24
AII	a-CiLSD1	*Ciona intestinalis*	XP002131150.1	12	12	16	105	0
AII	a-DlLSD1	*Delphinapterus leucas*	XP022407877.1	19	200	16	97	24
AII	a-DmLSD1	*Drosophila melanogaster*	NP001262100.1	2	162	17	104	0
AII	a-EcLSD1	*Equus caballus*	XP014592758.1	19	74	16	97	24
AII	a-GgLSD1	*Gallus gallus*	XP015153073.1	19	150	16	97	23
AII	a-HsLSD1	*Homo sapiens*	NP055828.2	20	176	16	97	24
AII	a-LaLSD1	*Loxodonta africana*	XP010593367.1	20	199	16	97	24
AII	a-MfLSD1	*Macaca fascicularis*	XP005544551.1	21	96	16	97	24
AII	a-MdLSD1	*Monodelphis domestica*	XP007491263.1	19	233	16	97	24
AII	a-MmLSD1	*Mus musculus*	NP598633.2	22	177	16	97	24
AII	a-SsLSD1	*Sus scrofa*	XP020948859.1	21	177	16	97	24
AII	a-TgLSD1	*Taeniopygia guttata*	XP002194853.1	19	87	16	97	24
AII	a-TcLSD1	*Tribolium castaneum*	XP008200898.1	6	91	16	96	12
AII	a-TcLSD1	*Tribolium castaneum*	XP008200898.1	6	91	16	96	12
AII	a-TtLSD1	*Tursiops truncatus*	XP019794105.1	19	197	16	97	24
PI	ch-OlLDL1	*Ostreococcus lucimarinus*	XP001422439.1	1	27	23	absent	56
PI	ch-OtLDL1	*Ostreococcus tauri*	XP003084447	0	99	37	absent	57
PIa	p-AgLDL1	*Aegilops tauschii*	XP020171831.1	0	139	21	absent	0
PIa	p-AmtLDL1	*Amborella trichopoda*	XP006853193.1	0	111	18	absent	138
PIa	p-AtLDL1	*Arabidopsis thaliana*	NP176471.1	0	162	21	absent	138
PIa	p-BdLDL1	*Brachypodium distachyon*	XP003570612.2	0	143	21	absent	134
PIa	p-BrLDL1	*Brassica rapa*	XP009113042.1	0	108	21	absent	273
PIa	p-CsLDL1	*Citrus sinensis*	XP006473753.1	0	124	21	absent	130
PIa	p-EsLDL1	*Eutrema salsugineum*	XP006391816.1	1	140	21	absent	144
PIa	p-GmLDL1a	*Glycine max*	KRH39215.1	0	141	22	absent	142
PIa	p-GmLDL1b	*Glycine max*	XP006584188.1	0	102	21	absent	140
PIa	p-GhLDL1	*Gossypium hirsutum*	XP016687848.1	0	110	44	absent	125
PIa	p-MaLDL1a	*Musa acuminata*	XP009391311.1	1	134	21	absent	129
PIa	p-MaLDL1b	*Musa acuminata*	XP018673947.1	4	127	23	absent	129
PIa	p-NtLDL1a	*Nicotiana tabacum*	XP016470400.1	0	151	21	absent	117
PIa	p-NtLDL1b	*Nicotiana tabacum*	XP016484866.1	0	151	21	absent	122
PIa	p-OsLDL1	*Oryza sativa*	XP015623199.1	0	167	21	absent	134
PIa	p-PvLDL1	*Phaseolus vulgaris*	XP007152780.1	0	99	21	absent	141
PIa	p-PtLDL1	*Populus trichocarpa*	XP002300664.1	0	122	21	absent	130
PIa	p-PrLDL1	*Prunus persica*	XP007225269.1	0	133	21	absent	37
PIa	p-RcLDL1	*Ricinus communis*	XP002529806.1	0	123	21	absent	127
PIa	p-SiLDL1	*Setaria italica*	XP004953964.1	0	161	21	absent	157
PIa	p-SlLDL1	*Solanum lycopersicum*	XP010327494.1	0	147	21	absent	121
PIa	p-StLDL1	*Solanum tuberosum*	XP006359789.1	0	147	21	absent	121
PIa	p-SbLDL1	*Sorgum bicolor*	XP002452532.2	1	160	21	absent	142
PIa	p-TcLDL1	*Theobroma cacao*	EOY15211.1	NA	110	21	absent	130
PIa	p-VvLDL1	*Vitis vinifera*	XP002265069.1	1	124	20	absent	8
PIa	p-ZmLDL1	*Zea mays*	XP008646288.1	0	160	21	absent	141
PIa	p-ZrLDL1	*Zostera marina*	KMZ68481.1	NA	181	23	absent	124
PIb1	p-AmtLDL2	*Amborella trichopoda*	XP006841765.1	3	56	18	absent	153
PIb1	p-AlLDL2	*Arabidopsis lyrata*	EFH61241.1	1	58	18	absent	143
PIb1	p-AtLDL2	*Arabidopsis thaliana*	NP187981.1	1	59	19	absent	150
PIb1	p-BdlDL2	*Brachypodium distachyon*	XP003573376.1	1	65	21	absent	167
PIb1	p-BrLDL2	*Brassica rapa*	XP009117559.1	1	55	18	absent	139
PIb1	p-CrLDL2	*Capsella rubella*	XP006297053.1	1	60	18	absent	138
PIb1	p-CsLDL2	*Citrus sinensis*	XP006464693.1	1	62	18	absent	146
PIb1	p-CusLDL2	*Cucumis sativus*	XP004150111.2	2	61	18	absent	269
PIb1	p-EsLDL2	*Eutrema salsugineum*	XP006407182.1	1	140	17	absent	147
PIb1	p-GmLDL2	*Glycine max*	XP003527270.1	2	60	18	absent	146
PIb1	p-HvLDL2	*Hordeum vulgare*	BAJ94616.1	NA	69	21	absent	157
PIb1	p-MtLDL2	*Medicago truncatula*	XP003600175.1	1	59	17	absent	153
PIb1	p-MaLDL2	*Musa acuminata*	XP009402613.1	1	63	18	absent	158
PIb1	p-NtLDL2a	*Nicotiana tabacum*	XP016461421.1	1	61	18	absent	167
PIb1	p-NtLDL2b	*Nicotiana tabacum*	XP016472582.1	1	60	18	absent	167
PIb1	p-OsLDL2	*Oryza sativa*	XP015650197.1	1	61	21	absent	165
PIb1	p-PvLDL2	*Phaseolus vulgaris*	XP007133132.1	NA	60	18	absent	0
PIb1	p-PtLDL2	*Populus trichocarpa*	XP002316929.2	2	60	18	absent	151
PIb1	p-PrLDL2	*Prunus persica*	XP007213630.1	1	60	18	absent	151
PIb1	p-RcLDL2	*Ricinus communis*	XP002533711.1	1	60	18	absent	153
PIb1	p-SiLDL2	*Setaria italica*	XP004972874.1	1	65	25	absent	164
PIb1	p-SlLDL2a	*Solanum lycopersicum*	XP010324354.1	4	64	18	absent	162
PIb1	p-SlLDL2b	*Solanum lycopersicum*	XP004244362.2	2	64	18	absent	158
PIb1	p-StLDL2	*Solanum tuberosum*	XP006360134.1	2	64	18	absent	162
PIb1	p-TcLDL2	*Theobroma cacao*	EOY12819.1	NA	60	18	absent	154
PIb1	p-VvLDL2	*Vitis vinifera*	XP002281860.3	1	60	18	absent	158
PIb1	p-ZmLDL2	*Zea mays*	XP008662882.1	1	62	21	absent	163
PIb2	p-MpFLD	*Marchantia polymorpha*	BAS31072.1	NA	113	18	absent	161
PIb2	p-PpFLDa	*Physcomitrella patens*	XP001761354.1	1	0	18	absent	147

PIb2	p-PpFLDb	*Physcomitrella patens*	XP001772025.1	1	0	18	absent	147
PIb2	p-SmFLD	*Selaginella moellendorffii*	XP002981558.1	2	27	18	absent	157
PIb2	p-AmtFLD	*Amborella trichopoda*	XP011624439.1	5	125	18	absent	168
PIb2	p-AlFLD	*Arabidopsis lyrata*	XP002882665.2	4	84	19	absent	262
PIb2	p-AtFLD	*Arabidopsis thaliana*	NP001326314.1	3	90	18	absent	67
PIb2	p-BdFLD	*Brachypodium distachyon*	KQJ84025.1	4	90	19	absent	181
PIb2	p-BnFLD	*Brassica napus*	XP013736108.1	4	108	21	absent	250
PIb2	p-BrFLD	*Brassica rapa*	XP009135110.1	4	140	26	absent	9
PIb2	p-CrFLD	*Capsella rubella*	XP006299707.1	4	77	19	absent	260
PIb2	p-EsFLD	*Eutrema salsugineum*	XP006407575.1	4	137	18	absent	251
PIb2	p-GmFLD	*Glycine max*	XP003520261.1	5	95	18	absent	231
PIb2	p-GhFLDa	*Gossypium hirsutum*	XP016748405.1	5	132	18	absent	224
PIb2	p-GhFLDb	*Gossypium hirsutum*	XP016698330.1	4	141	18	absent	204
PIb2	p-GhFLDc	*Gossypium hirsutum*	XP016731673.1	5	138	18	absent	234
PIb2	p-HvFLD	*Hordeum vulgare*	BAK05575.1	NA	84	19	absent	179
PIb2	p-MaFLD	*Musa acuminata*	XP009398021.1	2	119	18	absent	157
PIb2	p-OsFLD	*Oryza sativa*	BAS90468.1	4	96	19	absent	175
PIb2	p-PtFLDa	*Populus trichocarpa*	XP002315324.2	5	partial	18	absent	123
PIb2	p-PtFLDb	*Populus trichocarpa*	XP002311993.1	7	0	18	absent	325
PIb2	p-PrFLD	*Prunus persica*	XP020413288.1	5	132	18	absent	235
PIb2	p-RcFLD	*Ricinus communis*	EEF32788.1	4	123	18	absent	297
PIb2	p-SiFLD	*Setaria italica*	KQK98441.1	NA	91	18	absent	177
PIb2	p-SlFLDa	*Solanum lycopersicum*	XP004240184.1	7	87	18	absent	13
PIb2	p-SlFLDb	*Solanum lycopersicum*	XP004250050.1	5	135	18	absent	331
PIb2	p-StFLD	*Solanum tuberosum*	XP006361683.1	5	127	18	absent	332
PIb2	p-SbFLD	*Sorgum bicolor*	EES12649.1	4	91	19	absent	177
PIb2	p-VvFLD	*Vitis vinifera*	XP010658356.1	8	161	18	absent	327
PIb2	p-ZmFLD	*Zea mays*	NP001148070.1	1	91	19	absent	177
PIb2	p-ZrFLD	*Zostera marina*	KMZ63260.1	NA	151	18	absent	175
PII	p-AgLDL3	*Aegilops tauschii*	XP020158261.1	7	552	148	43	568
PII	p-AmtLDL3	*Amborella trichopoda*	XP011627240.1	8	781	168	50	2117
PII	p-AlLDL3	*Arabidopsis lyrata*	XP020873959.1	9	420	150	33	524
PII	p-AtLDL3	*Arabidopsis thaliana*	NP193364.5	10	380	150	33	524
PII	p-BdLDL3	*Brachypodium distachyon*	XP010234776.1	8	509	147	43	574
PII	p-BnLDL3	*Brassica napus*	CAB98166.1	9	343	150	37	187
PII	p-BrLDL3	*Brassica rapa*	XP009124513.1	9	337	150	37	524
PII	p-CrLDL3	*Capsella rubella*	XP006282993.1	12	386	150	37	524
PII	p-CsLDL3	*Citrus sinensis*	XP015384328.1	13	659	165	37	561
PII	p-CusLDL3	*Cucumis sativus*	XP004152762.1	8	633	149	10	575
PII	p-EsLDL3	*Eutrema salsugineum*	XP006414367.1	13	393	151	36	526
PII	p-GmLDL3a	*Glycine max*	XP014622663.1	8	584	152	33	588
PII	p-GmLDL3b	*Glycine max*	XP014625205.1	10	595	152	33	588
PII	p-GhLDL3a	*Gossypium hirsutum*	XP016751424.1	8	529	170	33	533
PII	p-GhLDL3b	*Gossypium hirsutum*	XP016666958.1	8	487	162	38	534
PII	p-GhLDL3c	*Gossypium hirsutum*	XP016694175.1	8	487	162	38	539
PII	p-MtLDL3a	*Medicago truncatula*	XP003589373.1	11	628	149	33	588
PII	p-MtLDL3b	*Medicago truncatula*	XP013462192.1	9	647	146	16	717
PII	p-MaLDL3	*Musa acuminata*	XP009389314.1	14	839	148	43	580
PII	p-NtLDL3	*Nicotiana tabacum*	XP016436973.1	5	679	187	38	541
PII	p-OsLDL3	*Oryza sativa*	XP015614418.1	8	519	158	39	575
PII	ch-OlLDL3	*Ostreococcus lucimarinus*	XP001416705.1	0	44	117	24	473
PII	ch-OtLDL3	*Ostreococcus tauris*	XP022838542.1	0	46	105	8	1506
PII	p-PpLDL3	*Physcomitrella patens*	XP001776061.1	19	451	264	94	618
PII	p-PtLDL3a	*Populus trichocarpa*	XP002307701.2	11	618	165	37	308
PII	p-PtLDL3b	*Populus trichocarpa*	XP002300727.2	8	610	171	38	295
PII	p-PrLDL3	*Prunus persica*	XP007225485.1	7	580	169	15	578
PII	p-RcLDL3	*Ricinus communis*	EEF52674.1	7	685	122	37	562
PII	p-SmLDL3	*Selaginella moellendorffii*	XP002993151.1	15	137	66	47	503
PII	p-SiLDL3	*Setaria italica*	XP004983837.1	9	467	161	43	564
PII	p-SlLDL3	*Solanum lycopersicum*	XP004238616.1	11	729	187	38	583
PII	p-StLDL3	*Solanum tuberosum*	XP006342013.1	9	730	187	38	583
PII	p-SbLDL3	*Sorgum bicolor*	XP002467596.1	9	477	167	43	571
PII	p-TcLDL3	*Theobroma cacao*	EOY14931.1	NA	647	170	39	531
PII	p-TuLDL3	*Triticum urartu*	EMS56268.1	NA	498	148	43	647
PII	p-VvLDL3	*Vitis vinifera*	XP010664765.1	9	795	164	39	587
PII	p-ZmLDL3	*Zea mays*	XP008657823.1	8	475	167	43	577
-	f-Sw1	*Schizosaccharomyces pombe*	NP595398.1	0	155	19	21	243
-	f-Sw2	*Schizosaccharomyces pombe*	NP592937.1	2	396	27	37	236
-	p-AtPAO1	*Arabidopsis thaliana*	NP196874.1					
