# Supplementary material for: The Four FAD-Dependent Histone Demethylases of Arabidopsis Are Differently Involved in the Control of Flowering Time
Source: Front Plant Sci. 2019 Jun 4;10:669. doi: 10.3389/fpls.2019.00669 (PMC6558185; doi:10.3389/fpls.2019.00669)
Supplement: Supplementary file 2 [file Table_2.pdf]

Supplementary Table 2. List of primers used in the present study. The sequence of all primers is shown from 5'end to 3' end.

**Preparation of constructs for *LDL/FLD::GFP-GUS* transgenic plants**	
PromLDL1-F	GGGGACAAGTTTGTACAAAAAAGCAGGCTGGGTCTTG ATGTCTCCACTACCTGCAG
PromtLDL1-R	GGGGACCACTTTGTACAAGAAAGCTGGGTAGCTCTAGC TCTGTGTGTGATGATGTGAGTG
PromLDL2-F	GGGGACAAGTTTGTACAAAAAAGCAGGCTACTAGTCT CTCTTTGCTTCTGTGTTGCTTTGTAG
PromLDL2-R	GGGGACCACTTTGTACAAGAAAGCTGGGTTTCTGGTCA ATTAAGTCACCAAATCACCTCC
PromFLD3-F	GGGGACAAGTTTGTACAAAAAAGCAGGCTGAAGAATA CACTGAAGGGAAAGGATG
PromFLD3-R	GGGGACCACTTTGTACAAGAAAGCTGGGTCTGCTCAAA ACTAGGGTTAGAGATTG
PromLDL3-F	GGGGACAAGTTTGTACAAAAAAGCAGGCTCTTCACCAT CCAACTTCCCATTTTC
PromLDL3-R	GGGGACCACTTTGTACAAGAAAGCTGGGTACCTATCTA CTTATTCCTTACACCC
**Genomic characterization of Arabidopsis *ldl/fld* mutants**	
LDL1-LP	CCCAGCTTTCTCAAGAAACCGCCG
LDL1-RP	GATGATGACGCTCTTCTTCTAGCAACTCTAAG
LDL2-LP	GATTACTGCGAAGATAATTCGCAACATGTAG
LDL2-RP	GTTTGCTCCTTATATTCCTGAAGAGGGTACT
FLD-LP	CTTATGCCTCGGGCCTTTGCAGATTGAGC
FLD-RP	GCACCAAAGAAACGAAGGAGAGGACG
LDL3-LP	CCCTCGGAAATTTTCATCTTCGCAGG
LDL3-RP	GACGATGATGCTGATGATGATGAACC
LBb1	GCGTGGACCGCTTGCTGCAACT
Lba1	GATGGTTCACGTAGTGGGCCATCGC
Lb3sail	GAATTTCATAACCAATCTCGATAC
**Characterization of *ldl/fld* Arabidopsis mutants by RT-PCR analysis**	
LDL1-F	CAACAGAGACTAAAGAAACCCGACC
LDL1-R	CGGCGGTTTCTTGAGAAAGCTGGG
LDL2-F	CCGAGGAGGAACAGGAG AAAAGTAAG
LDL2-R	AGTACCCTCTTCAGGAATATAAGGAGCAA
FLD-F	GCACCAAAGAAACGAAGGAGAGGACG
FLD-R	GCCGAATCTAAGAGACTACTACAATGTTTAGGAATC
LDL3-F	CAGCGCCAGGGTTTTTCTGTAACC
LDL3-R	TCTCAGCCGTTGAAGGCCATATTCT
UBQ5-F	GGAAGAAGAAGACTTACACC
UBQ5-R	AGTCCACACTTACCACAGTA
**qRT-PCR primers**	
LDL1-F	AGCTGCAAGACTGAATCGACA
LDL1-R	ACAACGTAGACGCAGCTGAA
LDL2-F	GCCCTGCCTTGGTTGCCTTA

Supplementary Table 2. (Continued)

LDL2-R	GGGTCAGGCACAACAACACCT
FLD-F	ACTGGTCGCAAGCTACCATCA
FLD-R	AATCCTTCTGATCATCTTCCGCTT
LDL3-F	TGGATCCTGGATTCGATGGGT
LDL3-R	CCTCAAGGCAAAGAGGTCACTC
FLC-F	CCGAACTCATGTTGAAGCTTGTTGAG
FLC-R	GTCGGAGATTTGTCCAGCAGGTGAC
FWA-F	GAAATTAGTCCAGGATTGTCTGC
FWA-R	TGAATTTTCTGCCACTTGTCC
MAF1-F	GGATCAGTGTTGTACCTCCTTCG
MAF1-R	TCCAATCCGTACATTCAGACAC
MAF2/3-F	CTCGCGCCAATGTGGAAAT
MAF2-R	AATTCTTTCGCATGAGCTTCTG
MAF3-R	AGGTACAACAAAGATCGGTAGCTT
MAF4-F	TGATTTGTAGTTCATAAGGAAAGC
MAF4-R	TTGATGCATCTCCTTGGATGAC
UBC21-F	CTGCGACTCAGGGAATCTTCTAA
UBC21-R	TTGTGCCATTGAATTGAACCC
