# Supplementary material for: The Four FAD-Dependent Histone Demethylases of Arabidopsis Are Differently Involved in the Control of Flowering Time
Source: Front Plant Sci. 2019 Jun 4;10:669. doi: 10.3389/fpls.2019.00669 (PMC6558185; doi:10.3389/fpls.2019.00669)
Supplement: Supplementary file 3 [file Data_Sheet_1.PDF]

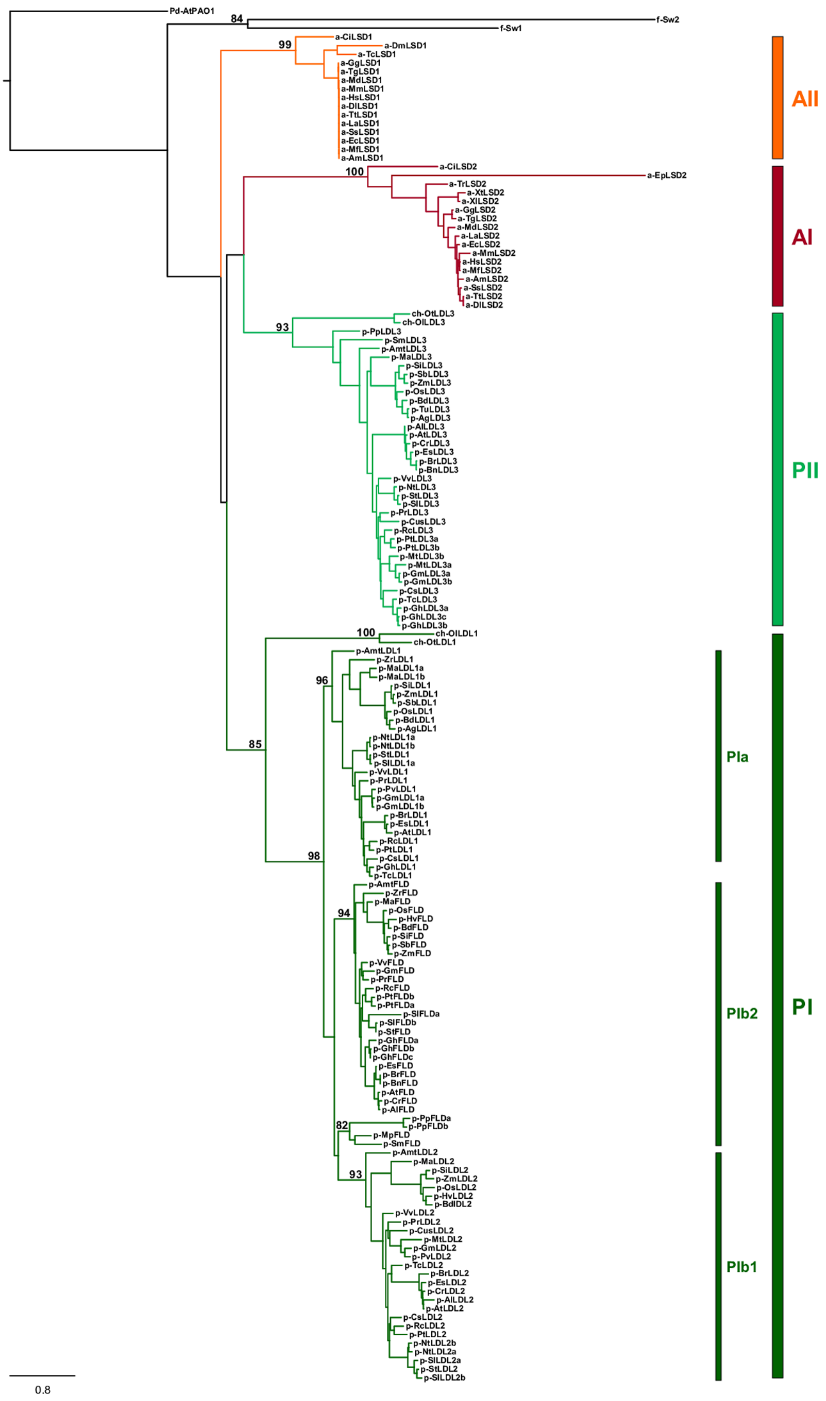

Supplementary Figure 1. Phylogenetic tree of amine oxidase domains of LDL/FLD homologues in representative plant species. Animal HsLSD1 and HsLSD2 homologues, as well as the two *Schizosaccharomyces pombe* homologues SWIRM1 and SWIRM2 (Nicolas et al., 2006) were also included in this analysis. Phylogenetic analyses were performed with the Maximum Likelihood method using RAXML v.8.2.10 (Stamatakis, 2014) with the PROTGAMMAJTT substitution model. Node support was evaluated with 1000 rapid bootstrap inferences. The sequence of the polyamine oxidase 1 of *A. thaliana* (AtPAO1; At5g13700; **Supplementary Table S1**) was used as outgroup.
