# Supplementary material for: The Four FAD-Dependent Histone Demethylases of Arabidopsis Are Differently Involved in the Control of Flowering Time
Source: Front Plant Sci. 2019 Jun 4;10:669. doi: 10.3389/fpls.2019.00669 (PMC6558185; doi:10.3389/fpls.2019.00669)
Supplement: Supplementary file 4 [file Data_Sheet_2.PDF]

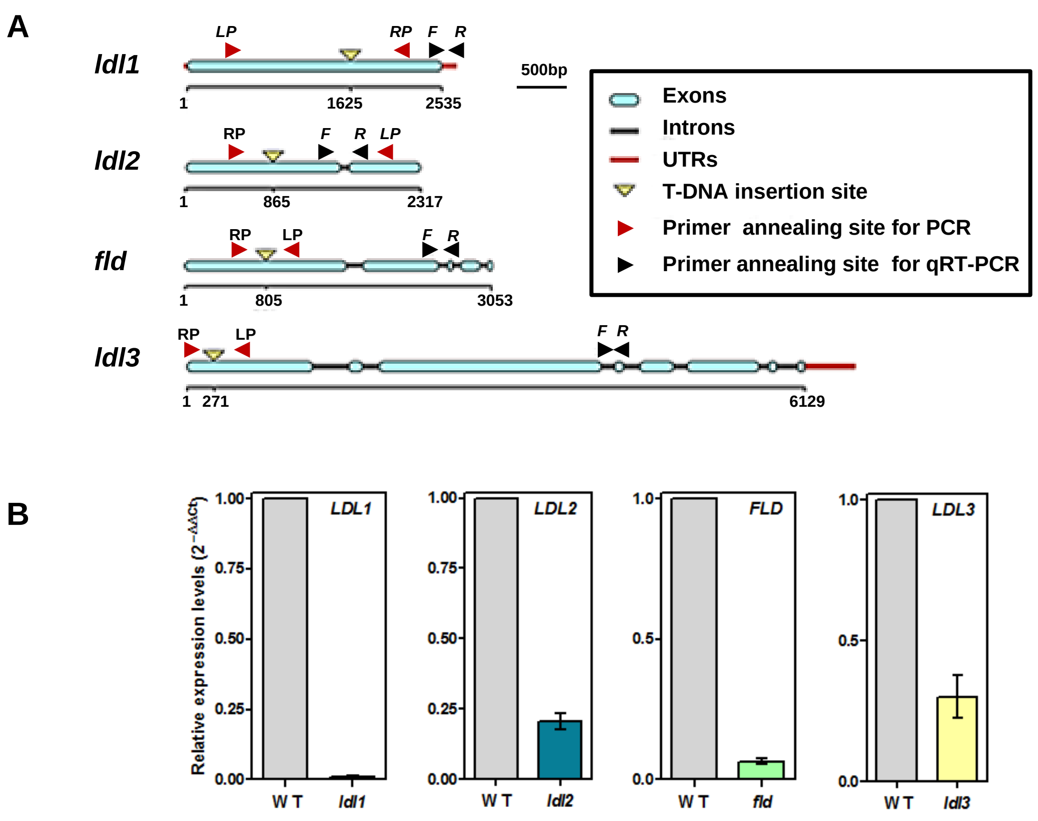

Supplementary Figure 2. Molecular characterization of loss-of-function Arabidopsis mutants for *LDL/FLDs*. (A) Graphic representation of *Arabidopsis LDL/FLD* genomic sequences. The position of the T-DNA insertions and the annealing sites of the primers used for mutant characterization by PCR and qRT-PCR is shown by arrowheads. Primer sequences are listed in **Supplementary Table 2**. The schematic representation is in scale. (B) Quantitative RT-PCR analysis of the four Arabidopsis *LDL/FLD* genes in the corresponding loss-of-function mutants as compared to the wild-type plants (WT). Bars represent standard error of three independent replicates.
