# Supplementary material for: The Four FAD-Dependent Histone Demethylases of Arabidopsis Are Differently Involved in the Control of Flowering Time
Source: Front Plant Sci. 2019 Jun 4;10:669. doi: 10.3389/fpls.2019.00669 (PMC6558185; doi:10.3389/fpls.2019.00669)
Supplement: Supplementary file 5 [file Data_Sheet_3.PDF]

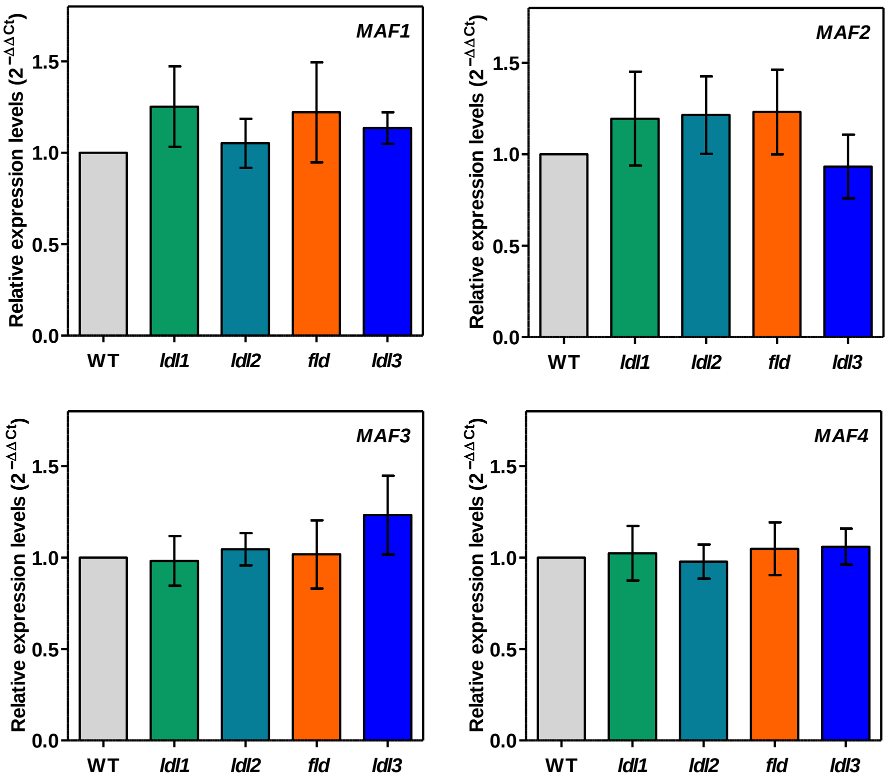

Supplementary Figure 3. Relative expression levels of *MAF1-MAF4* in Arabidopsis mutants for *LDL/FLDs*. Two-week-old seedlings of *ldl1*, *ldl2*, *fld*, *ldl3* Arabidopsis mutants and wild-type plants (WT) were analyzed for *MAF1*, *MAF2*, *MAF3* and *MAF4* expression levels by qRT-PCR. Numbers are mean values ±SE of three independent replicates. No statistically significant difference from WT plants was observed in *ldl/fld* mutants (one way ANOVA test, $p < 0.05$).
